# Chinese university students’ conceptions of feedback and the relationships with self-regulated learning, self-efficacy, and English language achievement

**DOI:** 10.3389/fpsyg.2022.1047323

**Published:** 2022-12-15

**Authors:** Shasha Lu, Liying Cheng, Saad Chahine

**Affiliations:** Faculty of Education, Queen’s University, Kingston, ON, Canada

**Keywords:** feedback, self-regulated learning, self-efficacy, English language achievement, Chinese university students

## Abstract

In China, under the influence of examination-driven culture and teacher-centered ways of learning, students’ self-regulated learning (SRL) capabilities, self-efficacy, and actual English proficiency are greatly hindered. Given this situation, the Chinese Ministry of Education has promulgated the use of formative assessment in the College English curriculum at the tertiary level since 2004. Feedback, as an integrated part of formative assessment, takes up the largest proportion of the Chinese College English classroom assessment and intends to facilitate SRL and learning. However, whether feedback could facilitate students’ SRL and learning has not been fully investigated in this context in China. Therefore, this study first explored how students self-reported their conceptions of feedback, SRL, and self-efficacy, and second, the relationships among these constructs and their English language achievement in the College English course. A questionnaire was used to collect data on students’ conceptions of feedback, SRL, self-efficacy, and self-perceived English language proficiency. Their English test scores as an indicator of English language achievement were also collected. A total of 538 participants from a university in Northern China participated in this study. Data were analyzed using descriptive statistics, exploratory factor analyses, Pearson correlation analyses, and multiple regression analyses. The results found that Chinese students from the College English course reported a high level of conceptions of teacher and peer feedback, SRL, and self-efficacy, yet a low level of Teacher/Peer Feedback Ignored. For the relationships among these variables, students’ conceptions of feedback contributed to SRL and self-efficacy. Besides, self-efficacy was found to be the strongest predictor for self-perceived English language proficiency and standardized English test scores, both indicators for English language achievement. From the theoretical perspective, this study addressed the research gap in the literature by examining four constructs together, that is, students’ conceptions of feedback, SRL, self-efficacy, and English language achievement within a university context in China. From the pedagogical angle, the results can also support teachers in their feedback practices to facilitate students’ SRL, self-efficacy, and learning.

## Introduction

In China, English education is put in a significant place. Millions of individuals learn English, but under the influence of the teacher-centered way of learning and examination-driven culture in the Chinese context (Cheng, 2008), students’ self-regulated learning (SRL) capabilities, their self-efficacy as a motivational factor, and their actual English proficiency are greatly hindered. Given this situation, the Chinese Ministry of Education recently initiated a series of educational reforms to facilitate learning, and quality assessment is one of these reforming targets (Chinese Ministry of Education, 2004, 2007). Formative assessment is advocated in higher education in China, for it measures students’ progress as a process instead of an outcome (Bulut et al., 2022). Feedback, as an integrated part of formative assessment, takes up the largest proportion of the College English classroom assessment in China (Jin, 2020). It intends to facilitate SRL and learning (Butler and Winne, 1995). However, whether feedback could facilitate students’ SRL capability and learning has not been fully investigated in the context of English learning in China, especially at the tertiary level. Therefore, this study explored the relationships among students’ conceptions of feedback, their SRL, self-efficacy, and English language achievement in the College English course in China.

## Literature review

Feedback was defined by Carless et al. (2011, p. 396) as “all dialogue to support learning in both formal and informal situations.” Teachers give feedback hoping that students will use it to regulate their learning and improve future performance (Brown et al., 2016). However, whether the feedback is effective is largely determined by students’ conceptions of it. Students’ conceptions of feedback refer to their beliefs about the nature and purpose of feedback (Brown et al., 2016). There are different classifications of feedback. Regarding its source, feedback can be internal and external. Internal feedback is generated by students themselves while external feedback is provided from external source, such as teachers, peers, and computers (Butler and Winne, 1995).

SRL is the degree that students are metacognitively, motivationally, and behaviorally active participating in their learning from the social cognitive perspective (Zimmerman, 1989). It is a cyclic process that consists of three self-regulatory phases: forethought, performance, and self-reflection (Zimmerman, 2013).

Self-efficacy, first put forward by Bandura in the social cognitive theory, refers to the belief in one’s capabilities to organize and regulate their action in prospective situations (Bandura, 1997). It is a motive not only for one’s performance but also for self-regulated and self-sustained learning (Bandura, 1986). A student’s SRL is motivated by one’s self-efficacy about successfully obtaining desired outcomes. Students’ self-efficacy and the ways to regulate their learning (SRL) together with their conceptions of feedback determine their learning outcome and academic achievement.

### Relationships among feedback, self-regulated learning, self-efficacy, and English language achievement

Feedback, SRL, and self-efficacy are closely correlated. The purpose of feedback is to improve SRL and performance (Butler and Winne, 1995). Self-efficacy and self-regulation are interconnected (Bandura, 1997). The cognitive aspects of self-regulation and motivational aspects of self-efficacy could not be separated from each other (Bandura, 1997). Some research indicates that certain types of feedback could promote self-efficacy and improve academic performance. Previous research demonstrates that feedback, SRL, and self-efficacy all improve students’ academic achievement. Self-efficacy was found to be the strongest predictor of academic performance in two meta-analyses (Robbins et al., 2004; Richardson et al., 2012). It was also found to be a strong predictor of language performance in the second language (L2) contexts (Raoofi et al., 2012). In addition, SRL could predict the language performance of college students (Zarei and Hatami, 2012). Feedback generated is also for students to improve their performance. However, few studies have yet to examine all four constructs together at the same time, especially in the English subject at the tertiary level in China. Some of the studies include two to three constructs of the four in a variety of settings and disciplines at different educational levels. Therefore, this literature review mainly focuses on the relationships between every two constructs of the three, that is, feedback, SRL, and self-efficacy, since they were all conceptualized to predict English language achievement.

#### Feedback and self-regulated learning

The feedback that teachers generate is intended to enable students to become self-regulated learners, especially in higher education such as the context of this study (Nicol and Macfarlane-Dick, 2006). More self-regulated learners tend to seek or use feedback more actively. Butler and Winne (1995) pointed out that feedback is an inherent catalyst and a prime determiner of SRL. They concluded that research on feedback and SRL should be studied together. Butler and Winne (1995) studied the impact of feedback on SRL in a model with a focus on the process of SRL. They argued that learners first interpret the task properties, and then set goals based on their interpretations. Tactics and strategies are selected to achieve goals and finally, learning products are generated. Through the monitoring processes, learners generate internal feedback and use it as evidence to modify their engagement by setting new goals, adjusting tactics and strategies, selecting better approaches, and establishing new procedures. The external feedback may “confirm, add to, or conflict with” (p. 248) learners’ existing knowledge and beliefs. Therefore, their existing knowledge and belief may be altered, thus impacting future self-regulation (Butler and Winne, 1995).

Many empirical studies also focused on the influence of different types of feedback on SRL. Two studies conducted on the subject of mathematics in China by Guo and Wei (2019) and Guo et al. (2019) confirmed the predictive power of certain types of feedback on students’ SRL. However, the results of a study by Zhu and Mok (2018) in Hong Kong revealed that the effect of students’ perceptions of teacher feedback on the SRL processes was weak after examining the possible predictors of the three processes of primary students’ SRL in mathematics.

The empirical research reviewed demonstrated that certain types of feedback can predict SRL in certain contexts, but they are not always positively correlated. The uncorrelated relationship between feedback and SRL in the empirical studies may be because the feedback as a variable in these studies focuses on the different types instead of the students’ conceptions of feedback. Different types of feedback are the characteristics of feedback, which could not guarantee students’ uptake. On the contrary, students’ conceptions of feedback are their attitude toward feedback, which could be more in line with their SRL. That is to say, students who believe in the usefulness of feedback may be more likely to use the feedback and reflect on, assess, and improve their performance, which are included in the SRL capabilities. Therefore, students’ conceptions of feedback are predictive of SRL. Besides, studies in different subjects and contexts may yield different results. The existing literature is mostly from primary and secondary levels (Zhu and Mok, 2018; Guo et al., 2019) and in Mathematics (Guo et al., 2019). Scarce studies are in the english as a foreign language (EFL) context at the tertiary level in China.

#### Feedback and self-efficacy

Some research indicates that certain types of feedback could promote self-efficacy and improve academic performance. Wang and Wu (2008) in Taiwan researched the role of feedback and self-efficacy in web-based learning from the social cognitive perspective. The result indicated that better quality feedback promoted students’ self-efficacy, as well as their performance. Brown et al. (2016) conducted a study in New Zealand on higher education investigating the relationships among students’ beliefs about the purpose of feedback, their self-regulation, academic self-efficacy, and academic achievement. The results showed that the factor “I use feedback” contributed to SRL, GPA, and self-efficacy, which indicated that students who believed in the positive functions of feedback tended to have higher SRL, self-efficacy, and academic performance.

Although the above two studies’ feedback positively influenced self-efficacy and academic performance, other studies revealed that feedback was not related to self-efficacy. An empirical study by Agricola et al. (2020) found that both verbal and written feedback did not improve self-efficacy, even though verbal feedback influenced students’ feedback perception more significantly than written feedback (Agricola et al., 2020).

In sum, there is an inconsistency in empirical studies concerning the relationships between feedback and self-efficacy. The inconsistent relationships in empirical studies between feedback and self-efficacy may arise from different contexts. Therefore, it is necessary to research the relationships between feedback and self-efficacy in different contexts. Besides, whether teachers’ feedback works effectively could be determined by students’ perceptions of it. Students’ perceptions of feedback are found to be the best predictor of students’ self-efficacy (van Dinther et al., 2014). Students who have positive perceptions of feedback are likely to have high self-efficacy (Agricola et al., 2020).

#### Self-efficacy and self-regulated learning

Self-efficacy and self-regulation are interconnected (Bandura, 1997). The cognitive aspects of self-regulation and motivational aspects of self-efficacy could not be separated from each other (Bandura, 1997). Besides, within Zimmerman’s (2013) SRL model, self-efficacy is an important component in the forethought phase of SRL, “because task analysis, goal setting, and strategic planning require personal initiative and persistence, high levels of key self-motivation beliefs/values” (p.143). According to Zimmerman (2013), proactive learners are motivated by self-efficacy, whereas reactive learners display lower levels of motivation.

Empirical research also indicated that self-efficacy and SRL are significantly connected. A study (2008) conducted in Taiwan at the higher education level revealed that self-efficacy positively predicted students’ use of learning strategies. Students with high self-efficacy tended to use more high-level learning strategies, which indicates that self-efficacy has a significant impact on SRL behaviors (Wang and Wu, 2008). One study (Kim et al., 2015) in Korea in the english as a second language (ESL) context investigated the relationships between learners’ different levels of self-efficacy and their SRL strategy use. The results revealed that self-efficacy is positively corrected with SRL strategy use.

Bai and Guo (2018) investigated the influences of the SRL strategy adoption on students’ self-efficacy in English writing in the EFL context in Hong Kong. One hundred and fifty-five students from fourth grade in a primary school in Hong Kong participated in this study. Two scales were used in this study. One scale was on five SRL strategies and the other was on self-efficacy in English writing. The results indicated that the use of planning and self-monitoring strategies of SRL was the greater predictor of students’ self-efficacy in English writing. The empirical literature revealed the reciprocal relationships between SRL and self-efficacy.

Previous research demonstrates that feedback, SRL, and self-efficacy are highly related. There are some inconsistencies in the relationships between feedback and SRL, and feedback and self-efficacy in the empirical studies reviewed so far. It may be caused by the construct feedback being measured in the reviewed literature, which is mostly different types of feedback emphasizing the characteristics of feedback itself, rather than students’ conceptions of feedback which emphasize more about students’ active role. Compared with the characteristics of feedback, how students believe the purpose of feedback is more in line with their SRL, self-efficacy, and English language achievement. However, few studies have yet to examine all four constructs together at the same time in the English subject at the tertiary level in China. Besides, there is an inconsistency in the literature reviewed so far, as to the direction of the relationship among the four constructs. Therefore, it is necessary to research the relationships among the four constructs in the EFL context at the tertiary level in China.

#### Conceptual framework and research questions

**FIGURE 1:**
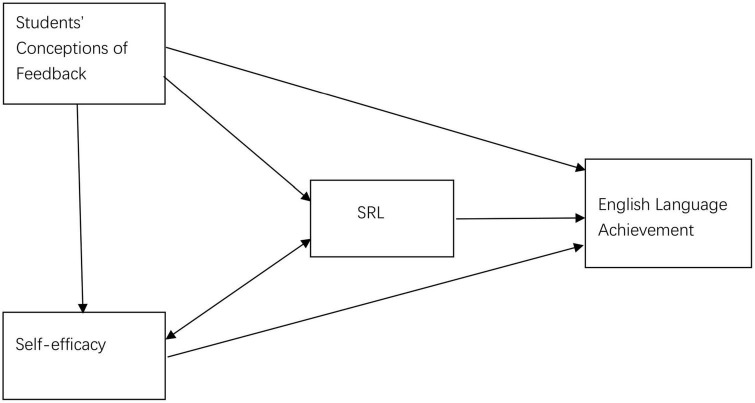
Schematic relationships among the four constructs.

Based on the previous research literature, we conceptualized a schematic relationship among students’ conceptions of feedback, SRL, self-efficacy, and English language achievement (see Figure 1) for this study. First, previous studies revealed that feedback is correlated with higher academic performance and it can raise students’ course grades (Hwang and Chang, 2011). In the context of the English subject, English language achievement serves as academic achievement of the outcome indicator. Therefore, students’ conceptions of feedback are hypothesized to predict English language achievement in this study. Second, the purpose of feedback is to facilitate SRL, especially in higher education (Nicol and Macfarlane-Dick, 2006), thus students’ SRL could be predicted by their conceptions of feedback. Students who have positive conceptions of feedback may have higher SRL capabilities. In addition, students’ conceptions of feedback could predict self-efficacy. Besides, self-efficacy and SRL strategy are closely correlated. Finally, English language achievement as an outcome variable could assess the predictive effect of the three highly correlated constructs, that is, students’ conceptions of feedback, SRL, and self-efficacy. We posed two specific research questions.

### Research questions

(1) How do Chinese students self-report their conceptions of feedback, SRL strategy use, and self-efficacy in the College English course in China?

(2) What are the relationships among students’ conceptions of feedback, their SRL strategy use, self-efficacy, and English language achievement in this context?

## Materials and methods

This study adopted a quantitative method to explore the relationships among students’ conceptions of feedback, SRL, self-efficacy, and English language achievement in the College English course in China based on Brown et al.’s (2016) study, since different research contexts may yield different results. A questionnaire was used to collect data about students’ conceptions of feedback, SRL, self-efficacy, and the students’ self-perceived English language proficiency scores. Their semester English test scores were also collected as an additional indicator for English language achievement.

### Research context

The study was conducted at a university in the Northern part of China. College English courses are required courses for all first- and second-year non-English major undergraduate students. There are four levels of College English courses (levels 1, 2, 3, and 4). First-year students normally take levels 1 and 2, and second-year students take levels 3 and 4. Students in years 3 and 4 normally do not have College English courses. Only the students who failed will retake the course. The assessments for College English courses are a combination of summative and formative assessments, with 40% of final scores from classroom assessments during the process of a whole semester and 60% of final scores from the unified summative assessments at the end of the semester. The classroom assessment includes speaking scores, listening scores, and scores from other forms of assignments and projects, such as presentations, role play, writing assignments, and so on, given by the course teacher. Students get feedback for the activities from their classroom teacher. The unified summative assessment includes reading, writing, translation, and vocabulary scores.

### Participants

Participants were undergraduates who were taking a College English course at a university in Northern China. A total of 538 students participated in this study.

### Instrument

A questionnaire consisting of five sections was used. Section one: Demographics consist of student number, gender, and major. Section two: Students’ conceptions of feedback were measured by the adapted version of the Student Conceptions of Feedback Questionnaire-III (Irving and Peterson, 2007). The adapted version consisted of 38 items. Section three: SRL strategies were measured by the Metacognitive Self-Regulation subscale of the Motivated Strategies for Learning Questionnaire (MSLQ) by Pintrich (1991). The subscale was selected because it focuses on the three phases of SRL (i.e., planning, monitoring, and reflecting) (Brown et al., 2016). It consists of 12 items. Section Four: Self-efficacy was also measured by the subscale of MSLQ (Pintrich, 1991). The Self-efficacy for Learning and Performance subscale of eight items was used. Section Five: Self-evaluation scale for English language proficiency, including five items, was used to assess participants’ self-perceived English proficiency as the indicator for English language achievement in their College English course. This scale included proficiencies in listening, speaking, reading, and writing, as well as overall proficiency. A 5-point Likert scale was adopted for all the questions.

### Data collection and data analysis

Before the study, ethics clearance was received in December 2021 from the General Research Ethics Board (GREB) at Queen’s University to ensure participants were aware of the risks and benefits of the research. The survey was in English and translated into Chinese by two graduate students in Canada whose first language is Chinese. A pilot study was conducted to check the accuracy of the translation, inviting five native Chinese speakers to complete the questionnaire and provide feedback for the translation.

The recruitment posters were sent on social media to invite students to participate. An online survey was used to collect data. As part of the participation, participants’ recent English test scores were obtained with the consent of the participants. At the beginning of the online survey, a question was asked to the participants whether they agreed to give consent to have access to their recent English test scores. If they agree, the following question would appear. Otherwise, the survey would stop. The latest English test scores were standardized to compare since the scores were from different classes. The standardized English test scores were used as an indicator of English language achievement in the College English course for this study.

Participants’ responses were entered into the Statistical Package for Social Sciences 27 (SPSS Version27) for analyses. To address the first research question, descriptive statistics of students’ conceptions of feedback, SRL, self-efficacy, and self-perceived English proficiency were calculated. Exploratory factor analyses were then used to identify the latent variables on the Students’ Conceptions of Feedback scale, SRL scale, Self-efficacy scale, and Self-perceived English Proficiency scale. Internal Consistency values were calculated for each factor. To address the second research question, Pearson correlation coefficients were calculated among all the variables, that is, students’ conceptions of feedback, SRL, self-efficacy, and self-perceived English proficiency. Subsequently, multiple regression analyses were conducted to explore the relationships with SRL, self-efficacy, and both English language achievement indicators (self-perceived English language proficiency and English test scores) as the dependent variables, respectively.

## Results

### Descriptive statistics

A total of 538 participants filled out the survey with no missing data. Students’ Conceptions of Feedback scale contained 38 items (Items 1–38). The highest means 4.47, 4.46, 4.39, 4.33, and 4.31 were from items 3, 1, 33, 16, and 22: “I look forward to getting feedback from my teacher on my work,” “Teachers give me trustworthy and honest feedback,” “I pay attention to feedback from my teacher,” “Feedback my teachers give me makes it clear how to improve,” and “I can trust feedback from my teacher.” The lowest means 1.65 and 1.72 were from items 17 and 12: “Teacher feedback doesn’t tell me anything useful” and “I ignore the comments teachers make about my work.” Standard Deviation (SD) for all the questions ranges from 0.82 to 1.38.

The SRL scale contains 12 items (Items 39–50). The highest mean of 4.03 was from item 41 “When I become confused about something I’m reading for this class, I go back and try to figure it out.” The lowest mean 2.94 was from item 46 “I often find that I have been reading for class but don’t know it was all about.” The range of SD was from 0.95 to 1.21.

The Self-efficacy scale contains eight items (Items 51–58). The highest mean 4.38 was from Item 56 “I expect to do well in this class,” which means participants overall agreement with this statement. The lowest mean 3.28 was from item 52 “I’m certain I can understand the most difficult material presented in the readings for this course.” The SD ranged from 0.88 to 1.19.

The Self-perceived English Proficiency scale consisted of five items (Items 59–63), self-perceived reading, writing, listening, speaking, and overall English proficiency. The result showed that the highest mean is from reading (*M* = 3.39), followed by overall English proficiency (*M* = 3.10), writing (*M* = 3.08), listening (*M* = 2.83), and speaking (*M* = 2.82).

### Factor analyses

Exploratory Factor Analysis (EFA) was not only to reduce the variables but also to understand the underlying structure of the measure for this study. A principal axis factoring (PAF) is used for extraction. Since these items are conceptually related, Oblique (Promax) was used for rotation. Four separate analyses were conducted, first on the Students’ Conceptions of Feedback scale, then on the SRL scale, Self-efficacy, and Self-perceived English Proficiency scale.

On the **Students’ Conceptions of Feedback scale**, the results of the Kaiser–Meyer–Olkin Measure (KMO) of Sampling Adequacy was 0.97. The value over 0.90 meant perfect sample size according to Field (2018). The Sig. value of Bartlett’s Test of Sphericity was 0.00. It is significant when *p* < 0.05, which indicates that the variables are correlated and the data are suitable for structure detection. An unrestricted EFA produced a 5-factor solution, this was then constrained to a 3-factor solution to better align with the scree-plot. These three factors explained 58.19% of the total variance with factors 1, 2, and 3 taking up 44.42, 10.36, and 3.41%, respectively. The items loading (see Table 1) on each factor were carefully examined, and the factors were labeled as (1) Teacher Feedback Motivates, (2) Peer Feedback Helps, and (3) Teacher/Peer Feedback Ignored. The Cronbach’s α of the Students’ Conceptions of Feedback is 0.95 and of the factor Teacher Feedback Motivates, Peer Feedback Helps, and Teacher/Peer Feedback Ignored are 0.96, 0.95, and 0.86, respectively, which indicates that all the 38 items were of high consistency. The Cronbach’s α and the EFA results suggest that data from this measure is reliable for further analysis.

**TABLE 1:** Factor loadings of the Students’ conceptions of feedback scale.

	Factor			Survey item number
	1	2	3	
**Teacher Feedback Motivates**				
I pay attention to feedback from my teacher	0.87			33
I can trust feedback from my teacher	0.83			22
I look at feedback to see what I did wrong	0.79			38
Good grades will help me get the job I want	0.77			35
Doing better than the expected or required standard is a good result	0.76			36
Teachers give me clear feedback	0.75			26
Feedback my teachers give me makes it clear how to improve	0.69			16
I know I have done well when my result is better than last time	0.69			32
The most useful feedback is from my teacher	0.67			8
I would rather get negative feedback in writing than out loud in front of the class	0.67			25
Teachers give me trustworthy and honest feedback	0.65			1
I look forward to getting feedback from my teacher on my work	0.65			3
I make active use of the feedback I get from my teacher	0.64			13
I actively use feedback to help me improve	0.63			28
I enjoy getting feedback	0.62			37
Feedback gives me information on how well I am doing	0.62			15
It is part of a teacher’s job to mark or grade my work	0.55			20
Feedback makes me try harder	0.54	0.34		31
Feedback is useless if we don’t get our work back quickly	0.41		0.31	23
I can usually predict just what my teacher will say about my work	0.39			34
**Peer Feedback Helps**				
Feedback from my classmates really helps me		0.92		14
Feedback from my classmates helps my learning		0.88		5
I look forward to getting feedback from my peers		0.88		29
I learn better when my friends comment on my work		0.82		2
I make active use of the feedback I get from my classmates		0.78		24
I can trust feedback from my peers		0.77		9
I do better when I work on something new with my friends		0.75		7
When I can’t understand something in		0.69		18
this class, I ask another student in the class for help				
Feedback changes the way I learn and study		0.57		21
I try to identify students in this class whom I can ask for help if necessary		0.56		11
I use feedback to set goals or targets for my next assessment	0.36	0.47		10
**Teacher/Peer Feedback Ignored**				
Teacher feedback doesn’t tell me anything useful			0.83	17
I ignore the comments teachers make about my work			0.80	12
Feedback does not tell me anything new			0.77	27
Teachers’ comments on my work are often hard to understand			0.69	30
Feedback is not necessary because I already know how well I am doing			0.68	6
I ignore bad grades or comments			0.67	19
I already know how good/poor my work is before I get any feedback			0.35	4

Extraction method: Principal axis factoring. Rotation method: Promax with Kaiser normalization. Rotation converged in 5 iterations. The items are arranged based on the loading from the highest to the lowest.

On the **SRL scale,** the value of Kaiser–Meyer–Olkin Measure of Sampling Adequacy was 0.93 and Bartlett’s Test of Sphericity was 0.00, indicating that the data set satisfied the conditions for EFA analysis. Two factors were identified on the SRL scale, with a cumulative variance of 54.23%. The two factors were named SRL Positive Behaviors and SRL Negative Behaviors (see Table 2). The Cronbach’s α of the SRL Scale is 0.89, and of the SRL Positive Behaviors and SRL Negative Behaviors are 0.93 and 0.58, respectively. Due to the low internal consistency, the research team removed the SRL Negative Behaviors.

**TABLE 2:** Factor loadings of the self-regulated learning scale.

	Factor		Survey item number
	1	2	
**SRL Positive Behaviors**			
When I study for this class, I set goals for myself in order to direct my activities in each study period.	0.83		49
I ask myself questions to make sure I understand the material I have been studying in this class.	0.83		44
I try to think through a topic and decide what I am supposed to learn from it rather than just reading it over when studying.	0.82		47
When studying for this course I try to determine which concepts I don’t understand well.	0.82		48
Before I study new course material thoroughly, I often skim it to see how it is organized.	0.74		43
If course materials are difficult to understand, I change the way I read the material.	0.73		42
If I get confused taking notes in class, I make sure I sort it out afterward.	0.72		50
When reading for this course, I make up questions to help focus my reading.	0.70		40
I try to change the way I study in order to fit the course requirements and instructor’s teaching style.	0.68		45
When I become confused about something I’m reading for this class, I go back and try to figure it out.	0.64		41
**SRL Negative Behaviors**			
During class time I often miss important points because I’m thinking of other things.		0.67	39
I often find that I have been reading for class but don’t know it was all about.		0.61	46

Extraction method: Principal axis factoring. Rotation method: Promax with Kaiser normalization. Rotation converged in 3 iterations. The items are arranged based on the loading from the highest to the lowest.

On the **Self-efficacy scale**, the KMO value was 0.94 and Bartlett’s Test of Sphericity was lower than 0.05, which were suitable for EFA. A one-factor structure was confirmed for the **Self-efficacy scale** (see Table 3), which accounted for 69.56%. This one factor was named Self-efficacy. The Cronbach’s α of the Self-efficacy scale is 0.95, indicating that the eight items had high internal consistency.

**TABLE 3:** Factor loadings of the self-efficacy scale.

	Factor	Survey item number
	1	
**Self-efficacy**		
I believe I will receive an excellent grade in this class	0.88	51
I’m certain I can understand the most difficult material presented in the readings for this course.	0.80	52
I’m confident I can understand the basic concepts taught in this course	0.87	53
I’m confident I can understand the most complex material presented by the instructor in this course.	0.87	54
I’m confident I can do an excellent job on the assignments and tests in this course	0.89	55
I expect to do well in this class	0.52	56
I’m certain I can master the skills being taught in this class	0.87	57
Considering the difficulty of this course, the teacher, and my skills, I think I will do well in this class	0.91	58

Extraction method: Principal axis factoring. 1 factors extracted. 4 iterations required. The items are arranged based on the loading from the highest to the lowest.

Finally, the KMO value was 0.83 and Bartlett’s Test of Sphericity was lower than 0.05 on the **Self-perceived English Proficiency scale**, which were suitable for EFA. A one-factor solution was also confirmed for the **Self-perceived English Proficiency scale** (see Table 4), accounting for 75.56% variance. This factor was named Self-perceived English Proficiency. The Cronbach’s α of the five items on the Self-perceived English Proficiency scale is 0.89, indicating the five items had high internal consistency.

**TABLE 4:** Factor loadings of the self-perceived English proficiency scale.

	Factor	Survey item number
	1	
**Self-perceived English proficiency**		
Self-perceived overall English proficiency	0.96	59
Self-perceived writing proficiency	0.77	0.60
Self-perceived reading proficiency	0.76	0.61
Self-perceived speaking proficiency	0.75	0.62
Self-perceived listening proficiency	0.73	0.63

Extraction method: Principal axis factoring. 1 factors extracted. 8 iterations required. The items are arranged based on the loading from the highest to the lowest.

### Correlation analyses

Pearson correlation was first conducted between Self-perceived English Proficiency and the standardized total English test scores to check whether students could accurately self-evaluate their English proficiency. Then, it was performed among all the potential variables to explore the relationships among students’ conceptions of feedback, SRL, self-efficacy, and English language achievement.

According to Plonsky (2015), the general benchmarks for interpreting the small, medium, and large effect size of correlations in L2 research were 0.25, 0.40, and 0.60, respectively. The results for the correlation analysis between students’ Self-perceived English Proficiency and standardized total English test scores showed that the standardized total English test scores was positively and significantly correlated with the Self-perceived English Proficiency (*r* = 0.23, *p* < 0.01), although the effect size was small.

A moderate to large relationship was found among students’ conceptions of feedback, SRL, self-efficacy, and English language achievement. The results (see Table 5) show that Self-efficacy was positively and significantly correlated with all the other factors. The highest coefficient of Self-efficacy was with SRL Positive Behaviors (*r* = 0.75, *p* < 0.01), followed by Teacher Feedback Motivates (*r* = 0.64, *p* < 0.01), Peer Feedback Helps (*r* = 0.60, *p* < 0.01), and Self-perceived English Proficiency (*r* = 0.54, *p* < 0.01).

**TABLE 5:** Correlation among all the variables.

			1	2	3	4	5	Standardized total English test scores	Self-perceived English proficiency
1	Teacher Feedback Motivates	Pearson correlation	1					0.030	0.33**
		Sig. (2-tailed)						0.485	<0.001
2	Peer Feedback Helps	Pearson correlation	0.82**	1				−0.035	0.32**
		Sig. (2-tailed)	<0.001					0.413	<0.001
3	Teacher/Peer Feedback Ignored	Pearson Correlation	−0.14**	0.16	1			−0.08	0.24**
		Sig. (2-tailed)	0.001	<0.001				0.074	<0.001
4	SRL Positive Behaviors	Pearson Correlation	0.71**	0.70**	0.14**	1		0.03	0.44**
		Sig. (2-tailed)	<0.001	<0.001	0.001			0.493	<0.001
5	Self-efficacy	Pearson Correlation	0.64**	0.60**	0.15**	0.75**	1	0.15**	0.54**
		Sig. (2-tailed)	<0.001	<0.001	<0.001	0.000		<0.001	<0.001

**Correlation is significant at the 0.01 level (2-tailed).

SRL Positive Behaviors were positively and significantly correlated with Self-efficacy (*r* = 0.75, *p* < 0.01), Teacher Feedback Motivates (*r* = 0.71, *p* < 0.01), Peer Feedback Helps (*r* = 0.70, *p* < 0.01), and Self-perceived English Proficiency (*r* = 0.44, *p* < 0.01).

The results also indicated that the factors Teacher Feedback Motivates and Peer Feedback Helps from Students’ Conceptions of Feedback scale were positively and significantly correlated with SRL Positive Behaviors, Self-efficacy, and Self-perceived English Proficiency.

Finally, Self-perceived English Proficiency was positively and significantly correlated with Self-efficacy (*r* = 0.54, *p* < 0.01), SRL Positive Behaviors (*r* = 0.44, *p* < 0.01), Teacher Feedback Motivates (*r* = 0.33, *p* < 0.01), Peer Feedback Helps (*r* = 0.32, *p* < 0.01), and Teacher/Peer Feedback Ignored (*r* = 0.24, *p* < 0.01). The standardized total English test scores were only significantly correlated with Self-efficacy (*r* = 0.15, *p* < 0.01), but the effect size was small.

The mean of all the independent variables are as follows: Teacher Feedback Motivates (*M* = 4.15), Peer Feedback Helps (*M* = 3.90), Teacher/Peer Feedback Ignored (*M* = 2.24), SRL Positive Behaviors (*M* = 3.68), and Self-efficacy (*M* = 3.70).

### Regression analyses

To answer research question 2, multiple regression analyses were conducted to explore the contributive power of students’ conceptions of feedback on SRL strategy use and self-efficacy. Additionally, stepwise regression analyses were performed to explore the extent students’ conceptions of feedback, SRL strategy use, and self-efficacy contributed to their English language achievement.

#### Predictive power of students’ conceptions of feedback on self-regulated learning strategy use and self-efficacy

In the regression, the three identified variables on students’ conceptions of feedback were entered as independent variables, and with the SRL Positive Behaviors were entered as a dependent variable.

As shown in Table 6, students’ conceptions of feedback explained 54.6% variance of the SRL Positive Behaviors. The two factors, Teacher Feedback Motivates (β = 0.41, *p* < 0.01) and Peer Feedback Helps (β = 0.36, *p* < 0.01) from the Students’ conceptions of feedback, were significant predictors for SRL Positive Behaviors. Teacher/Peer Feedback Ignored (β = 0.03, *p* = 0.399) was not found to be a significant predictor for SRL Positive Behaviors.

**TABLE 6:** Multiple regression analysis of Students’ conceptions of feedback on SRL positive behaviors.

*R* ^2^	Adjusted *R*^2^	F (df1, df2)	Variable	Standardized coefficients β	*t*	*P*	95% CI for B
0.741	0.546	216.565** (3, 534)	Teacher	0.41**	8.16	<0.001	[0.41, 0.67]
			Peer	0.36**	7.23	<0.001	[0.27, 0.47]
			Ignored	0.03	0.85	0.399	[0.03, 0.08]

**p < 0.01. Teacher, Teacher Feedback Motivates; Peer, Peer Feedback Helps; Ignored, Teacher/Peer Feedback Ignored.

To explore the predictive power of students’ conceptions of feedback on Self-efficacy, students’ conceptions of feedback variables were treated as the independent variables and the Self-efficacy variable as the dependent variable. For the regression model with the dependent variable Self-efficacy, students’ conceptions of feedback explained 42.7% of the variance (see Table 7). Teacher Feedback Motivates (β = 0.44, *p* < 0.01) and Peer Feedback Helps (β = 0.23, *p* < 0.01) were significant predictors for Self-efficacy. Teacher/Peer Feedback Ignored (β = 0.05, *p* = 0.106) was not a significant predictor for Self-efficacy.

**TABLE 7:** Multiple regression analysis of Students’ conceptions of feedback on self-efficacy.

*R* ^2^	Adjusted *R*^2^	F (df1, df2)	Variable	Standardized coefficients β	*t*	*P*	95% CI for B
0.427	0.423	132.45** (3, 534)	Teacher	0.44**	7.84	<0.001	[0.47, 0.79]
			Peer	0.23**	4.02	<0.001	[0.13, 0.37]
			Ignore	0.05	1.62	0.106	[−0.01, 0.12]

**p < 0.01. Teacher, Teacher Feedback Motivates; Peer, Peer Feedback Helps; Ignored, Teacher/Peer Feedback Ignored.

#### Predictive power of self-efficacy, self-regulated learning, and students’ conceptions of feedback on the English language achievement

Stepwise regression analyses were performed with feedback, SRL, and Self-efficacy variables as the independent variables, with Self-perceived English Proficiency as the dependent variable.

The results (see Table 8) indicated that Teacher Feedback Motivates, Peer Feedback Helps, and SRL Positive Behaviors did not contribute to students’ Self-perceived English Proficiency. In Model 1, Self-efficacy was the significant predictor for students’ Self-perceived English Proficiency. After adding the variable Teacher/Peer Feedback Ignored, Model 2 was also significant. This indicated that 31.8% of the Self-perceived English Proficiency could be explained by Self-efficacy and Teacher/Peer Feedback Ignored.

**TABLE 8:** Stepwise regression analysis of Students’ self-perceived English proficiency.

Model	Adjusted *R*^2^	F change (df1, df2)	Variable	Standardized coefficients β	*t*	*P*	95% CI for B
1	0.292	222.84** (1, 536)	SE	0.54**	14.93	<0.001	[0.40, 0.52]
2	0.318	21.17** (1, 535)	SE	0.52**	14.33	<0.001	[0.38, 0.50]
			Ignored	0.17**	4.60	<0.001	[0.08, 0.20]

**p < 0.01. SE, Self-efficacy; Ignored, Teacher/Peer Feedback Ignored.

The same procedures were performed with standardized total English test scores entered as the dependent variable. The results showed (see Table 9) that the total variance explained by Self-efficacy, Peer Feedback Helps, and Teacher/Peer Feedback Ignored in the standardized total English test scores was 4.9%. The results indicated all the constructs explained a small variance in the standardized total English test scores. Among the three constructs, Self-efficacy was the only positive predictor, accounting for a comparatively larger proportion of variance in the standardized total English test scores.

**TABLE 9:** Stepwise regression analysis of standardized total English test scores.

Model	Adjusted *R*^2^	F change (df1, df2)	Variable	Standardized coefficients β	*t*	*P*	95% CI for B
1	0.020	12.17** (1, 536)	SE	0.15**	3.49	<0.001	[0.07, 0.26]
2	0.043	13.54** (1, 535)	SE	0.27**	5.03	<0.001	[0.18, 0.41]
			Peer	−0.19	−3.68	<0.001	[−0.36, −0.11]
3	0.049	4.28** (1, 534)	SE	0.27**	5.18	<0.001	[0.19, 0.42]
			Peer	−0.18**	−3.49	<0.001	[−0.35, −0.10]
			Ignored	−0.09*	−2.07	0.039	[−0.19, −0.01]

**p < 0.01, *p < 0.05. SE, Self-efficacy; Peer, Peer Feedback Helps; Ignored, Teacher/Peer Feedback Ignored.

## Discussion

This study addressed two research questions, which explored first, how students self-reported their conceptions of feedback, SRL, and self-efficacy, and second, the relationships among these constructs and their English language achievement in the College English course.

### Unique findings

Research Question 1: How students self-reported their conceptions of feedback is answered by the first subtitle **High conceptions of feedback** below. Further, how students self-reported their SRL and self-efficacy also from the first research question is answered under the second subtitle **Students’ conceptions of feedback contributed to high self-regulated learning and high self-efficacy**. The second subtitle together with the third subtitle **Self-efficacy and English language achievement** answered the second research question with regard to the relationships among the four constructs.

#### High conceptions of feedback

This study’s first unique finding is that students from this EFL context had high conceptions of feedback regardless of its source, that is, teacher feedback or peer feedback. However, Chinese students trusted teacher feedback more than peer feedback.

The Students’ Conceptions of Feedback scale yielded three factors in this study: Teacher Feedback Motivates, Peer Feedback Helps, and Teacher/Peer Feedback Ignored. According to Oxford (1990), a mean in the range of 3.5–5.0 is categorized as a high level, 2.5–3.4 medium level, and 1.0–2.4 low level. The mean of Teacher Feedback Motivates and Peer Feedback Helps was 4.15 and 3.90 out of 5, respectively, which indicated that students formed a high-level conception of feedback based on Oxford’s (1990). It suggested that participants found the teacher and peer feedback useful and they made active use of the feedback. The results echoed a study (Gan, 2020) conducted on Chinese university EFL students. Both studies found that the participants thought teachers’ feedback helpful, and they made generally active use of teacher feedback as part of a process of SRL. But these results contrasted with students’ dissatisfaction with feedback in general education (Carless and Boud, 2018). On the other hand, the mean of this Teacher/Peer Feedback Ignored was 2.24 out of 5, which is a low-level conception of feedback based on Oxford’s classification of the level of learners’ self-reported questionnaire data. This indicated that most students from this context did not think they should ignore teacher/peer feedback.

Besides, compared with Teacher Feedback Motivates, Peer Feedback Helps had a lower mean, which indicated that Chinese students trusted teacher feedback more than peer feedback. The factor structure of the Students’ Conceptions of Feedback scale from this study also confirms this result. The Students’ Conceptions of Feedback scale from Brown et al. (2016) previous study, also at the tertiary level, yielded more factors than it did in the current study. Only three factors (Teacher Feedback Motivates, Peer Feedback Helps, and Teacher/Peer Feedback Ignored) were yielded from the Students’ Conceptions of Feedback scale in this study, while Brown et al. (2016) had an additional three factors (Active use of feedback, Enjoyment, and Meet expectations). This means that in the current study, the three additional factors from Brown et al. (2016) were included mostly in the factor Teacher Feedback Motivates. This result, therefore, indicated that participants from this context found teacher feedback more enjoyable and they made active use of it.

One of the reasons is that students from this context treated teachers as experts, trusting their feedback (Yang et al., 2006; Zhan, 2019). For example, a study revealed that Chinese EFL students used more teacher feedback than peer feedback to improve L2 writing at tertiary education in China because teachers were more professional, experienced, and trustworthy than their peers, but the importance of peer feedback was also recognized by the participants (Yang et al., 2006). However, other studies questioned the quality of peer feedback because peers were unwilling to give negative comments about their classmates, and were more likely to emphasize the strengths of their classmates’ work (Yang et al., 2006; Zhan, 2019).

#### Students’ conceptions of feedback contributed to high self-regulated learning and high self-efficacy

The second unique finding of this study is that students’ conceptions of feedback contributed to the self-reported high SRL and high self-efficacy in their performance in the College English course. In addition, SRL and Self-efficacy were significantly and highly correlated in this study.

The mean of SRL Positive Behaviors (*M* = 3.68) and Self-efficacy (*M* = 3.70) suggested that students self-identified as self-regulated learners with high self-efficacy in the College English course. This is consistent with the previous study (Guo and Wei, 2019) conducted on secondary math students in China, in that students generally reported a medium to high level of SRL strategies use and a high level of motivation. Students in that study can be generally identified as self-regulated learners in Math. The high level of SRL in the College English course in the current study could also be attributed to the development of students’ SRL in the Curriculum Reform of China (Ministry of Education of the People’s Republic of China, 2014).

Students’ conceptions of feedback were conceptualized to predict SRL and self-efficacy, and this study confirmed this claim. The factors Teacher Feedback Motivates and Peer Feedback Helps of the students’ conceptions of feedback were found to predict SRL Positive Behaviors and Self-efficacy. Teacher Feedback Motivates was the strongest predictor in this prediction, followed by Peer Feedback Helps.

The results are consistent with the previous research (Brown et al., 2016) that conceptions of feedback were in line with SRL. However, the inconsistency with Brown et al.’s (2016) lies in whether the factor Teacher Feedback Motivates predicted SRL. In Brown et al. (2016), Teacher Feedback had no contributions to SRL, and Actively Use Feedback (+ Enjoy) was the strongest predictor for SRL, while in this study, Teacher Feedback Motivates was found to be the strongest predictor. This may result from the factor structure of this study. As analyzed, the items from the Actively Use Feedback (+ Enjoy) factor of students’ conceptions of feedback in Brown et al.’s (2016) mostly went to the factor Teacher Feedback Motivates in this study. This indicated that Chinese students in the College English course found teacher feedback enjoyable and used feedback actively. Therefore, it is not surprising to find the factor Teacher Feedback Motivates was the strongest predictor for SRL in this study because the participants who used feedback actively had higher SRL levels. In addition, Chinese students from the College English course with a higher conception of teacher feedback were more self-efficacious and more self-regulated learners. This may result from the better quality of teacher feedback. Teachers in this context may target to develop SRL and self-efficacy in students when giving feedback.

For self-efficacy, in Brown’s study, only the factor Actively Use Feedback + Enjoy contributed to Self-efficacy, while Teacher/Tutor Feedback and Peers Help did not contribute to it. In this study, the Chinese students from the College English course who held positive conceptions of teacher feedback and peer feedback had higher levels of self-efficacy. A study in Taiwan (Wang and Wu, 2008) echoed this study that students who received elaborative feedback contributed to their self-efficacy significantly compared to those who did not.

Teacher/Peer Feedback Ignored was not found to be a significant predictor for both SRL Positive Behaviors or Self-efficacy. The results are consistent with the previous study that the factor Ignore Feedback had no predictions for SRL or self-efficacy in Brown’s study.

Besides, SRL and Self-efficacy were significantly and highly correlated (*r* = 0.75, *p* < 0.01) in this study. Most empirical studies used self-efficacy as the predictor for SRL. Students who had higher self-efficacy used more and higher levels of SRL strategies (Wang and Wu, 2008; Kim et al., 2015). While other researchers used SRL as the predictor for self-efficacy. Students who used more SRL strategies presented a higher level of self-efficacy in English writing (Bai and Guo, 2018). In the current study, SRL and Self-efficacy were highly correlated. Their relationships between them could be reciprocal (Wang and Bai, 2017). This is also consistent with Zimmerman’s model, in which self-efficacy is perceived as a component of SRL.

In summary, there was a strong alignment between SRL and self-efficacy, students’ conceptions of feedback and SRL, as well as students’ conceptions of feedback and self-efficacy. The results showed that students who reported having higher conceptions of feedback, both from teachers and peers, also reported taking up self-regulated strategies in their studies and having high self-reported self-efficacy within the Chinese higher education context.

#### Self-efficacy and English language achievement

The third unique finding of this study is that Self-efficacy was found to be the biggest contributor to English language achievement. There are two indicators for English Language Achievement in this study, self-perceived English proficiency and the standardized English test scores. For the self-perceived English proficiency scores, the biggest significant predictor was Self-efficacy, followed by Teacher/Peer Feedback Ignored. Teacher Feedback Motivates, Peer Feedback Helps, and SRL Positive Behaviors were not significant predictors for Self-perceived English Proficiency. Judge et al. (1998) defined self-evaluations as conclusions that individuals hold about themselves, and self-efficacy is considered an indicator of the core self-evaluations. Individuals who have higher self-efficacy may self-evaluate higher since self-efficacy is self-perceived capability to achieve specific tasks (Pajares, 1996). The second predictor for Self-perceived English Proficiency was Teacher/Peer Feedback Ignored. At first, the results seemed contradictory, but if participants ignored teacher or peer feedback, they may have higher self-efficacy, since self-efficacy is the belief in one’s capabilities to organize and regulate their action in prospective situations (Bandura, 1997). Besides, if participants ignore feedback from external sources, such as teachers and peers, they may have a higher level of self-regulating information responses (Brown et al., 2016). The students who rejected feedback may have higher self-efficacy which was the biggest predictor for self-perceived English proficiency.

However, this study could not differentiate whether students had higher levels of self-efficacy or they may self-evaluate higher if they ignored feedback from external sources. Additionally, we do not know the reasons why these students ignored feedback. Further research is needed to probe deeper into the reasons why Chinese students from College English classes ignored the feedback and how to make feedback easier to be used.

For the standardized English test scores, Self-efficacy was also found to be the biggest positive predictor, followed by Peer Feedback Helps and Teacher/Peer Feedback Ignored, but they were negative predictors. This indicated students who believed Peer Feedback Helps or Teacher/Peer Feedback Ignored had a lower standardized English test score. Teacher Feedback Helps and SRL Positive Behaviors were not significant predictors for the standardized English test scores, either. There were no direct relationships between SRL Positive Behaviors and standardized English test scores, which is consistent with Brown’s study as well as the meta-analysis, that it, is self-efficacy rather than SRL that predict academic performance (Richardson et al., 2012; Brown et al., 2016). In addition, Teacher Feedback Motivates did not contribute directly to the standardized English test scores in this study, while in Brown’s study, Teacher Feedback and Peer Feedback were found to predict academic performance negatively. But in another study by Irving et al. (2008), using this Students’ Conceptions of Feedback scale on secondary students in the New Zealand, the researchers found that Teacher Feedback contributed greatly to mathematics achievement (β = 0.40) positively. There are several possibilities to explain this unpredictive power of Teacher Feedback Motivates on English language achievement in this study.

Students from the College English course had positive conceptions of feedback, but they may not have the opportunity to apply the feedback they received to the next task or assignment (Brown et al., 2016). If students did not act on the feedback, even if they believed feedback was useful, the feedback they received would not automatically contribute to their English language achievement. As in the study by Butler and Winne (1995), external feedback works together with students’ internal responses to it. If the external feedback is not acted on, the internal responses do not happen, thus not contributing to English language achievement.

Another possibility is that students in this context regarded their teachers as authorities, so they were passively guided by the feedback, seldom negotiating, discussing, or clarifying with their teachers, thus weakening the improving function of teacher feedback (Zhan, 2019).

In addition, teachers’ feedback targeted specific tasks of this course, that is, presentations, discussions, and so on, which may be irrelevant to the end-of-term exams. There have been doubts about whether test scores could truly reflect students’ actual English abilities, especially the end-of-term exams, which does not serve selective purposes and may contain the scores of efforts. Therefore, teacher feedback may not contribute to the end-of-term test score.

The factor Teacher/Peer Feedback Ignored is hypothesized to decrease English language achievement. However, the results showed that it positively predicted Self-perceived English Proficiency and negatively contributed to English test scores. It could be understood that students who ignored the teacher and peer feedback may have higher self-efficacy, thus having a higher self-evaluation of their English proficiency. Moreover, as expected, Teacher/Peer Feedback Ignored negatively contributed to the standardized English test scores, which indicated that students who ignored feedback from teachers or peers may have a lower end-of-term English test score. The findings echoed the results of a study conducted in the Chinese university English lessons that found most students perceived more encouraging functions than improving functions of feedback (Zhan, 2019). The encouraging functions of feedback contributed to self-efficacy, while the least recognized improving function of feedback was demonstrated in the unpredictive or even negative power of feedback on the standardized test scores.

Peer Feedback Helps had a negative predictive power on the standardized English test scores, but positively contributed to SRL and Self-efficacy, which is consistent with Brown’s findings. According to him, peers are not the official markers for the class, and relying on them may not improve their test scores (Brown et al., 2016).

To sum up, this study found that Chinese students from the College English course reported a high level of conceptions of teacher and peer feedback, SRL, and self-efficacy, yet a low level of Teacher/Peer Feedback Ignored. For the relationships among these variables, students’ conceptions of feedback contributed to SRL and self-efficacy. Besides, self-efficacy was found to be the strongest predictor for self-perceived English language proficiency and standardized English test scores, both indicators for English language achievement.

## Implications and conclusion

This study found that students who reported having positive conceptions of feedback have higher self-efficacy and higher SRL capabilities, no matter whether the feedback is from teachers or peers. Thus, high-quality feedback should be provided by external sources such as teachers and peers to improve students’ conceptions of feedback, thus improving their SRL and self-efficacy. Feedback, as a key part of assessment literacy, should be taught to both teachers and students to improve feedback quality in higher education. Peers should also be trained to give effective feedback (Tian and Zhou, 2020). Feedback should not only target specific tasks but also improve SRL, self-efficacy, and overall performance.

Further research is needed to explore qualitatively the reasons why Chinese students from the College English course ignore teacher and peer feedback and how to make feedback easier to put into use, further guiding pedagogical practices. The sample of this study was only from one university in China, thus the implications of the results are limited to this sample. Future research could collect data from an equal sampling of a range of Chinese universities to validate the results of this study.

Despite the limitation of the study, the findings of this study have both theoretical and pedagogical implications. From the theoretical angle, this study adds to the research literature by investigating Chinese students’ conceptions of feedback, their SRL, and self-efficacy levels for the College English course. Besides, it explored the relationships among students’ conceptions of feedback, their SRL, self-efficacy, and English language achievement in this context.

From the pedagogical angle, self-efficacy was found to be the strongest predictor for self-perceived English proficiency scores and standardized English test scores. Therefore, students’ self-efficacy should be put in a significant place to be cultivated by teachers and students themselves in the College English course. Teachers need to adjust their assessment practices in the classroom to develop Students’ self-efficacy.

## Data availability statement

The raw data supporting the conclusions of this article will be made available by the authors, without undue reservation.

## Ethics statement

The studies involving human participants were reviewed and approved by the General Research Ethics Board at Queen’s University. A Written Letter of Information was provided, with completion of the survey representing consent.

## Author contributions

SL, LC, and SC contributed to conception and design of the study. SL performed the statistical analysis and wrote the first draft of the manuscript. LC and SC guided the manuscript writing, contributed to manuscript revision, read, and approved the submitted version. All authors contributed to the article and approved the submitted version.

## References

[B1] Agricola B. T. Prins F. J. Sluijsmans D. M. A. (2020). Impact of feedback request forms and verbal feedback on higher education students’ feedback perception, self-efficacy, and motivation. *Assess. Educ.* 27 6–25. 10.1080/0969594X.2019.1688764

[B2] Bai B. Guo W. (2018). Influences of self-regulated learning strategy use on self-efficacy in primary school students’ English writing in Hong Kong. *Read. Writ. Q.* 34 523–536. 10.1080/10573569.2018.1499058

[B3] Bandura A. (1986). The explanatory and predictive scope of self-efficacy theory. *J. Soc. Clin. Psychol.* 4 359–373. 10.1521/jscp.1986.4.3.359

[B4] Bandura A. (1997). *Self-efficacy : The exercise of control.* New York, NY: W.H. Freeman.

[B5] Brown G. T. L. Peterson E. R. Yao E. S. (2016). Student conceptions of feedback: Impact on self-regulation, self-efficacy, and academic achievement. *Br. J. Educ. Psychol.* 86 606–629. 27612004 10.1111/bjep.12126

[B6] Bulut O. Shin J. Cormier D. C. (2022). Learning analytics and computerized formative assessments: An application of Dijkstra’s shortest path algorithm for personalized test scheduling. *Mathematics* 10:2230.

[B7] Butler D. L. Winne P. H. (1995). Feedback and self-regulated learning: A theoretical synthesis. *Rev. Educ. Res.* 65 245–281. 10.3102/00346543065003245

[B8] Carless D. Boud D. (2018). The development of student feedback literacy: Enabling uptake of feedback. *Assess. Eval. High. Educ.* 43 1315–1325. 10.1080/02602938.2018.1463354

[B9] Carless D. Salter D. Yang M. Lam J. (2011). Developing sustainable feedback practices. *Stud. High. Educ.* 36 395–407. 10.1080/03075071003642449

[B10] Cheng L. (2008). The key to success: English language testing in China. *Lang. Test.* 25 15–37. 10.1177/0265532207083743

[B11] Chinese Ministry of Education (2004). *大学英语课程教学要求 (试行) [College English curriculum requirements (for trial implementation)].* Beijing: Tsinghua University Press.

[B12] Chinese Ministry of Education (2007). *大学英语课程教学要求 [College English curriculum requirements]* Beijing: Foreign Language Teaching and Research Press.

[B13] Field A. (2018). *Discovering statistics using IBM SPSS statistics*, 5th Edn. Thousand Oaks, CA: SAGE.

[B14] Gan Z. (2020). How learning motivation influences feedback experience and preference in Chinese university EFL Students. *Front. Psychol.* 11:496. 10.3389/fpsyg.2020.00496 32265798 PMC7100336

[B15] Guo W. Wei J. (2019). Teacher feedback and students’ self-regulated learning in mathematics: A study of Chinese secondary students. *Asia Pac. Educ. Res.* 28 265–275. 10.1007/s40299-019-00434-8

[B16] Guo W. Lau K. L. Wei J. (2019). Teacher feedback and students’ self-regulated learning in mathematics: A comparison between a high-achieving and a low-achieving secondary schools. *Stud. Educ. Eval.* 63 48–58.

[B17] Hwang G. J. Chang H. F. (2011). A formative assessment-based mobile learning approach to improving the learning attitudes and achievements of students. *Comput. Educ.* 56 1023–1031. 10.1016/j.compedu.2010.12.002

[B18] Irving S. E. Peterson E. R. (2007). *Student conceptions of feedback (SCOF) inventory (version 3) [Measurement instrument].* Auckland: University of Auckland.

[B19] Irving S. E. Peterson E. R. Brown G. T. L. (2008). “Feedback and academic achievement: The relationship between students’ conceptions of feedback and achievement,” in *Proceedings of the 6th biennial conference of the international test commission*, Liverpool. 10.1111/bjep.12126

[B20] Jin Y. (2020). 大学英语评价与测试的现状调查与改革方向 [Current practices and reform agendas of college english testing and assessment]. *Foreign Lang. World* 41, 2–9.

[B21] Judge T. A. Locke E. A. Durham C. C. Kluger A. N. (1998). Dispositional effects on job and life satisfaction: The role of core evaluations. *J. Appl. Psychol.* 83:17. 10.1037/0021-9010.83.1.17 9494439

[B22] Kim D. H. Wang C. Ahn H. S. Bong M. (2015). English language learners’ self-efficacy profiles and relationship with self-regulated learning strategies. *Learn. Individ. Differ.* 38 136–142. 10.1016/j.lindif.2015.01.016

[B23] Ministry of Education of the People’s Republic of China (2014). *On comprehensively deepening curriculum reform, and views of implementing the fundamental task of morality education.* Beijing: Chinese Government.

[B24] Nicol D. J. Macfarlane-Dick D. (2006). Formative assessment and self-regulated learning:A model and seven principles of good feedback practice. *Stud. High. Educ.* 31 199–218. 10.1080/03075070600572090

[B25] Oxford R. L. (1990). *Language learning strategies: What every teacher should know.* New York, NY: Newbury House/Harper & Row.

[B26] Pajares F. (1996). Self-efficacy beliefs in academic settings. *Rev. Educ. Res.* 66 543–578. 10.3102/00346543066004543

[B27] Pintrich P. R. (1991). *A manual for the use of the motivated strategies for learning questionnaire (MSLQ).* Ann Arbor, MI: University of Michigan.

[B28] Plonsky L. (2015). “Statistical power, *p* values, descriptive statistics, and effect sizes: A “back-to-back” approach to advancing quantitative methods in L2 research,” in *Advancing quantitative methods in second language research*, ed. Plonsky L. (New York, NY: Routledge), 23–45.

[B29] Raoofi S. Tan B. H. Chan S. H. (2012). Self-efficacy in second/foreign language learning contexts. *Engl. Lang. Teach.* 5 60–73. 10.5539/elt.v5n11p60

[B30] Richardson M. Abraham C. Bond R. (2012). Psychological correlates of university students’ academic performance: A systematic review and meta-analysis. *Psychol. Bull.* 138 353–387. 10.1037/a0026838 22352812

[B31] Robbins S. B. Lauver K. Le H. Davis D. Langley R. Carlstrom A. (2004). Do psychosocial and study skill factors predict college outcomes? A meta-analysis. *Psychol. Bull.* 130:261. 10.1037/0033-2909.130.2.261 14979772

[B32] Tian L. Zhou Y. (2020). Learner engagement with automated feedback, peer feedback and teacher feedback in an online EFL writing context. *System (Linköping)* 91:102247. 10.1016/j.system.2020.102247

[B33] van Dinther M. Dochy F. Segers M. Braeken J. (2014). Student perceptions of assessment and student self-efficacy in competence-based education. *Educ. Stud.* 40 330–351. 10.1080/03055698.2014.898577

[B34] Wang C. Bai B. (2017). Validating the instruments to measure ESL/EFL learners’ self-efficacy beliefs and self-regulated learning strategies. *TESOL Q.* 51 931–947. 10.1002/tesq.355

[B35] Wang S. L. Wu P. Y. (2008). The role of feedback and self-efficacy on web-based learning: The social cognitive perspective. *Comput. Educ.* 51 1589–1598. 10.1016/j.compedu.2008.03.004

[B36] Yang M. Badger R. Yu Z. (2006). A comparative study of peer and teacher feedback in a Chinese EFL writing class. *J. Second Lang. Writ.* 15 179–200. 10.1016/j.jslw.2006.09.004

[B37] Zarei A. A. Hatami G. (2012). On the relationship between self-regulated learning components and L2 vocabulary knowledge and reading comprehension. *Theory Pract. Lang. Stud.* 2 1939–1944. 10.4304/tpls.2.9.1939-1944

[B38] Zhan Y. (2019). Conventional or sustainable? Chinese university students’ thinking about feedback used in their english lessons. *Assess. Eval. High. Educ.* 44 973–986. 10.1080/02602938.2018.1557105

[B39] Zhu J. Mok M. M. C. (2018). Predicting primary students’ self-regulated learning by their prior achievement, interest, personal best goal orientation and teacher feedback. *Educ. Psychol.* 38 1106–1128. 10.1080/01443410.2018.1497775

[B40] Zimmerman B. J. (1989). A social cognitive view of self-regulated academic learning. *J. Educ. Psychol.* 81 329–339. 10.1037/0022-0663.81.3.329

[B41] Zimmerman B. J. (2013). From cognitive modeling to self-regulation: A social cognitive career path. *Educ. Psychol.* 48 135–147. 10.1080/00461520.2013.794676

